# Multiresistant Bacterial Pathogens Causing Bacterial Pneumonia and Analyses of Potential Risk Factors from Northeast Ethiopia

**DOI:** 10.1155/2021/6680343

**Published:** 2021-03-08

**Authors:** Tewodros Dessie, Mohabaw Jemal, Minwuyelet Maru, Moges Tiruneh

**Affiliations:** ^1^Amhara Public Health Institute, Dessie Branch, P.O. Box 686, Dessie, Ethiopia; ^2^University of Gondar, College of Medicine and Health Sciences, School of Biomedical and Laboratory Sciences, Department of Medical Microbiology, P.O. Box 196, Gondar, Ethiopia

## Abstract

**Background:**

Pneumonia is the most common cause of morbidity and mortality in developing countries, mostly caused by different species of bacterial pathogens. Hence, patient management needs awareness of the pathogens and antimicrobial susceptibility testing (AST). This study was aimed to assess the type of bacterial isolates and their antimicrobial susceptibility patterns among pneumonia suspected patients at Dessie Referral Hospital, Northeast Ethiopia. Potential risk factors were also assessed to apply preventive measures accordingly.

**Materials and Methods:**

A cross-sectional study design was employed among pneumonia suspected patients from February to April 2020 at Dessie Referral Hospital. Sociodemographic characteristics and associated risk factors were collected using a pretested questionnaire, and clinical data were extracted by reviewing medical records. Sputum specimens were collected and inoculated into chocolate agar, blood agar, mannitol salt agar, and MacConkey agar which are then incubated at 35°C or 37°C for 24–48 hours. Bacterial species were identified based on Gram stain, colony characteristics, and biochemical techniques. The data were entered in to Epi-Info version 7.1.5 and analyzed with SPSS software version 20. *p* value <0.05 at 95% CI was considered as statistically significant.

**Results:**

A total of 406 sputum specimens were collected and cultured, among which 157 (38.7%) were positive for different bacterial pathogens. The predominant pathogens were *Klebsiella pneumoniae* (28.0%), *Streptococcus pneumoniae* (24.8%), *Staphylococcus aureus* (18.5%), and *Pseudomonas aeruginosa* (14.0%). Majority of the isolates exhibited resistance to ampicillin with 81.5% followed by penicillin with 75.9% and amoxicillin-clavulanate with 61.2%. Multivariable logistic regression showed a significant association of culture positivity with older age (AOR = 2.43, CI: 1.12–5.28, *p* value = 0.025), cigarette smoking (AOR = 4.67, CI: 2.39–9.20, *p* value <0.001), and alcohol use (AOR = 5.58, CI: 3.14–9.92, *p* value <0.001). Resistance to ampicillin and penicillin was associated with repeated prescription and use.

**Conclusions:**

This study found high prevalence of bacterial pneumonia in the study area, and high rate of bacterial resistance was observed in ampicillin, penicillin, and amoxicillin-clavulanate. Repeated prescriptions and use of antimicrobials were significantly independent factors of bacterial resistance. Therefore, patient management needs identification of bacteria by routine culture with antimicrobial susceptibility testing.

## 1. Introduction

Pneumonia, supported with a clinical and/or radiological consolidation of the lungs, is an inflammation (acute or chronic) of the lung parenchyma [1]. This inflammation is produced mostly by microorganisms, particularly bacteria [2, 3]. The extracellular bacteria responsible for pneumonia include *S. pneumoniae*, *H*. *influenzae*, and *Staphylococcus aureus*, while *Pseudomonas aeruginosa* and other Gram-negative bacilli are infrequent causes of the disease. Moreover, the “atypical” intracellular bacteria responsible for this disease, without being cultured by routine culture methods, include *Mycoplasma pneumoniae*, *Chlamydophila pneumoniae*, and *Legionella pneumophila* [4]. Pneumonia can be transmitted by different ways, inhalation of droplets (e.g., *C. pneumoniae* and *M. pneumoniae*), environmentally (*L. pneumophila*), and microaspiration of a potential pathogen after colonization of the nasopharynx (e.g., *S. pneumoniae*, *H. influenzae*, or *S. aureus*) [4]. In all ways, the infection of the pulmonary parenchyma leads the bronchioles and alveoli to be filled with inflammatory exudates. It leads to a decrease of carbon dioxide and oxygen exchange between blood and the lungs and causes respiratory scarcity, making it hard for infected persons to breathe [5, 6].

To make selection of initial antimicrobial therapies easier, the disease is usually classified as community-acquired pneumonia (CAP) or healthcare-associated pneumonia (HCAP) [7]. There is high prevalence of multidrug-resistant (MDR) pathogens in healthcare settings, HCAP patients receiving broad empiric therapies [7]. Moreover, the clinical presentations for both types of the disease may range from mild pneumonia (fever and productive cough) to severe pneumonia (respiratory distress and sepsis) [8, 9]. In developing countries, pneumonia is the most common cause of illness in adults visiting hospitals [10]. The disease also affects children and elders in a high proportion [11]. This indicates that pneumonia is among the leading causes of morbidity and mortality in all age groups [12]. Globally, the increase in the burden of bacterial pneumonia with age needs more attention to the disease in the future [13]. In addition, pneumonia causes crisis in terms of health expenses and days of work lost [14]. As indicated by several studies, high incidence, admission, and mortality rates of bacterial pneumonia are contributing factors for both health crisis and economic burdens [13, 15, 16]. Moreover, severe pneumonia can cause long-term complications such as bronchiectasis which may persist to chronic obstructive lung disease [17].

Key factors for the development of the disease reported by several studies were aging, smoking, alcoholism, chronic obstructed pulmonary diseases (COPD), hospitalization for long periods, chronic disease, immunodeficiency, contact with contaminated hospital materials, and exposure to antibiotics for a long period and viral infections of the respiratory tract, as it compromises the respiratory tract and results in bacterial colonization and infection [18–22].

Globally, community-acquired pneumonia (CAP) causes approximately 31.1 per 100000 deaths in people less than 19 years [23]. The global annual incidence rate of CAP in the age group of 18–39 and ≥75 years is 6/1000 and 34/1000, respectively. Among all cases, 20–40% of patients need admission, from which 5–10% of them are admitted to intensive care units due to severe complications such as septic shock, extrapulmonary organ dysfunction, and acute respiratory distress syndrome (ARDS). Moreover, the overall mortality among adults from CAP is 6–15%, which magnifies the importance of identifying and treating patients with this disease [24–26]. In the United States, 5 million to 10 million patients are treated for community-acquired pneumonia (CAP) annually [27], with greater than $10 billion medical costs [28]. In Africa, the mortality rate of adult patients was in the range of 6% and 15% [29]. In South Africa, CAP was the fifth largest killer, accounting 3.9% of all deaths [30]. Based on previous reports, the prevalence of CAP in Ethiopia was in the range of 40–48% [12, 31–34].

In developing countries, bacterial pneumonia is treated usually empirically; by medical history and physical examinations, the etiologic agent is rarely identified [35] and results in high prevalence of multidrug-resistant (MDR) pathogens [7]. Hence, identifying the most common bacterial pathogens and their antimicrobial susceptibility patterns timely and accurately is key to reduce morbidity and mortality due to the disease [36], as the empiric treatment is based on severity of pneumonia, the prevalence, and local antimicrobial resistance patterns [22, 37, 38].

Therefore, awareness about the etiology of pneumonia is fundamental for appropriate patient management. In Northeast Ethiopia, however, there was scarce of studies showing the real burden of bacterial pneumonia among suspected patients. In addition, there are scarce of studies about antimicrobial susceptibility patterns of the common bacterial agents for pneumonia. Therefore, this study aimed to assess the prevalence of bacterial pathogens, their antimicrobial susceptibility patterns, and associated risk factors among patients suspected for bacterial pneumonia attending Dessie Referral Hospital, Northeast Ethiopia.

## 2. Materials and Methods

### 2.1. Study Design and Setting

A hospital-based cross-sectional study was conducted from February to April 2020 in Dessie Referral Hospital, Northeast Ethiopia. The hospital had around 500 beds with annual ambulatory cases of around 14400; hence, it was purposefully selected to conduct this study. All pneumonia suspected patients visiting the hospital were used as the source population, and all patients aged ≥5 years who were clinically suspected for bacterial pneumonia were included in the study. Patients who were under antimicrobial treatment within the last 14 days during data collection were excluded from the study. Pneumonia prevalence of 40.3% from the previous study was used to determine the number of study participants [1].

### 2.2. Sample Size and Sampling Technique

A total of 406 study participants were proposed and systematically recruited.

### 2.3. Data Collection

Data related to sociodemography and risk factors for bacterial pneumonia were collected by pretested and structured questionnaire through face-to-face interview. Gene Xpert results, initial diagnosis, and nutritional status of study participants were collected by reviewing medical records. Pneumonia suspected patients were clinically selected based on chest pain, shortness of breath, cough with sputum production, fever, night sweats, shaking chills, hemoptysis, and altered mental state (confusion). Sputum specimens were collected in a sterile, disposable, leak proof, and wide-mouthed container with tight-fitting lid. To reduce the number of commensals, the purulent parts of the sputum specimens were washed in about 5 ml of sterile physiological saline. To keep pathogens (such as *S. pneumoniae* and *H. influenzae*) alive, the washed sputum specimens were inserted to a cotton-wool swab which then inserted in containers of Amies transport medium; then, all specimens were transported with cold box to Amhara Public Health Institute (APHI), Dessie Branch Bacteriology Laboratory for culture and antimicrobial susceptibility testing.

### 2.4. Bacterial Isolation and Identification

All specimens received for culture were evaluated macroscopically followed by microscopic inspection by Gram stain before culture analysis began. Thus, sputum specimens with at least 25 polymorph-nuclear leukocytes and <10 epithelial cells per low power field and >10 bacteria per high-powered field were processed for culture [39–41].

The sputum specimens appropriate for culture were inoculated into blood agar plate (BAP), MacConkey agar plate (MAC), mannitol salt agar (MSA), and chocolate agar plate (CHO). Subsequently, BAP, MAC, and MSA were incubated at 37°C for 18–24 hours, while CHO (in humid, 5% CO_2_ atmosphere) was incubated for 18–24 hours at 35°C–37°C. All the plates were examined for growth after 24 hours; the plates without growth were further incubated for up to 48 hrs. The colonies were subcultured on BAP and MAC for further identification. Bacterial species were identified based on Gram stain, colony characteristics (such as size, shape, pigmentation, and color) zones of hemolysis, and other biochemical characteristics. *Streptococcus pneumoniae* was identified by catalase and optochin (5 *μ*g) sensitivity tests, while *S. aureus* isolates were confirmed by catalase, coagulase, and the mannitol fermentation tests. Chocolate agar, enriched with factor V (NAD) and factor X (hemi), was used to enhance the growth of *H. influenzae.* Gram-negative isolates were inoculated onto different biochemical tests such as motility, indole, urea, lysine decarboxylase, triple sugar iron agar, and citrate utilization tests for identification [42].

### 2.5. Antimicrobial Susceptibility Testing

Antimicrobial susceptibility testing (AST) for bacterial isolates was performed according to Clinical and Laboratory Standards Institute' (CLSI) recommendations [43]. The applied discs were tetracycline (TE_30 *μ*g), erythromycin (E_15 *μ*g), penicillin (P_10 *μ*g), ceftriaxone (CRO_30 *μ*g), doxycycline (DA_30 *μ*g), trimethoprim-sulphamethoxazole (TMP-SMX_1.25 + 23.75 *μ*g), ciprofloxacin (CIP_5 *μ*g), gentamicin (CN_10 *μ*g), ampicillin (AMP_10 *μ*g), imipenem (IMP_10 *μ*g), cefepime (PEP_30 *μ*g), amoxicillin/clavulanic acid (AMC_20/10 *μ*g), piperacillin/tazobactam (TZP_100/10 *μ*g), amikacin (AK_30 *μ*g), cefuroxime (CXM_30 *μ*g), ceftazidime (CAZ_30 *μ*g), chloramphenicol (CAF_30 *μ*g), meropenem (MER_10 *μ*g), aztreonam (AZT_30 *μ*g), oxacillin (OXA_1 *μ*g), and cefoxitin (CXT_30 *μ*g).

A young culture growth of bacterial suspensions was prepared by picking parts of similar colonies with a sterile wire loop in which these suspensions were adjusted to McFarland 0.5 turbidity standard. The bacterial suspensions in a sterile broth were incubated up to 2 hours to allow the bacteria to reach their log-phase in growth. Then, inoculums were swabbed on to Muller–Hinton agar. After drying the agar for 3–5 minutes, the antimicrobial impregnated disks were placed with sterile forceps on the agar surface in such a way that each disk was placed at least 24 mm away from each other to avoid the overlapping zone of inhibition. After placing the discs, the plates were allowed to stand for 30 minutes to help the antimicrobial to be dissolved in the media. Following inverting and incubating for 24 hours at 37°C, the plates were read for the diameter of zone of inhibition. The susceptibility patterns were graded as sensitive, intermediate, and resistant. Muller–Hinton agar (MHA) supplied with 5% sheep blood was used for *S. pneumoniae*, while Muller–Hinton agar containing 1.0% hemoglobin and 1.0% IsoVitaleX supplement (CHOC-MHA) was used for *H. influenzae* [42].

### 2.6. Laboratory Quality Control

Manufacturer instructions and bacteriological standard procedures were followed strictly throughout the whole technical processes including culture media preparation, inoculation, and AST testing. The sterility of culture media was checked by incubating 5% of the batch at 35–37°C overnight and was evaluated for possible contamination. The standard reference bacterial strains such as *S. aureus (*ATCC®25923), *H. influenzae* (ATCC® 49247), *E. coli (*ATCC® 35218), and *S. pneumoniae* (ATCC® 49619) were used as a quality control [43].

### 2.7. Data Quality Control

Training was given for the data collector about data collection procedures and interview techniques. To assure the quality of the data, a pretested, structured questionnaire was used for data collection. The questionnaire was objective-based and logically sequenced. It was checked daily by the principal investigator for its completeness.

### 2.8. Data Analysis

Data were checked for completeness, cleaned, coded, and entered in to Epi-Info version 7.1 software and then exported to SPSS version 20 for analysis. Frequency, proportions, and summary statistics were used to describe study participants in relation to relevant variables. Bivariate and multivariate logistic regression analyses were carried out to identify the association between bacterial pathogens, antimicrobial resistance, and possible risk factors. Odds ratio and *p* value were used to assess the presence and degree of association. *p* value <0.05 at 95% CI was considered as statistically significant.

### 2.9. Ethical Approval and Consent to Participate

Ethical clearance was obtained from University of Gondar, College of Medicine and Health Sciences Institutional Review Board. Informed written consent was obtained from the study participants after explaining the purpose and objective of the study. The laboratory results from the study participants were communicated to their physicians for appropriate patient management. Data from all patients were kept confidential.

### 2.10. Limitations of the Study

This study did not consider the “atypical” intracellular bacteria, such as *Mycoplasma pneumoniae*, *Chlamydophila pneumoniae*, and *Legionella pneumophila*, since they cannot be cultured by routine culture methods. This study did not also consider the anaerobic bacteria (*Prevotella* spp., *Fusobacterium* spp., and *Clostridium* spp.) as routine culture methods are mostly aerobic. Hence, it underestimates the actual prevalence in the study area. This study did not include serotyping techniques to *Haemophilus influenzae* and *Streptococcus pneumoniae.* Characterization of methicillin-resistant *Staphylococcus aureus* (MRSA) was not performed.

## 3. Results

### 3.1. Sociodemographic and Clinical Data Characteristics

This study was conducted among 406 pneumonia suspected patients, of which 221 (54.4%) were males and 249 (61.3%) were urban dwellers with 158 (38.9%) participants unable to read and write (Table 1). The median age of study participants was 45.0 with a range of 10–95 years. Among study participants, 131 (32.3%) were smokers and 158 (38.9%) were alcohol consumers.

Among all participants, 43 (10.6%) were HIV positives and 39 (9.6%) had active TB cases at the time of data collection. As indicated in Table 1, asthma, diabetes, and hypertension comorbidities accounted 6%, 5%, and 3%, respectively. The nutritional status of children (*n* = 30) was assessed by mid-upper arm circumferences (MUAC) and body mass index (BMI), all of which (*n* = 30, 100%) showed normal nutritional status.

### 3.2. Prevalence of Bacterial Pathogens

Bacterial isolates were identified from 157 (38.7 %) participants, of which 155 (98.7%) were identified as community-acquired pneumonia (CAP) while 2 (1.3%) were hospital-acquired pneumonia (HAP). Gram-negative isolates accounted 89 (56.7%), while the rest 68 (43.3%) were Gram-positive. The frequently isolated pathogens were *Klebsiella pneumoniae*, *Streptococcus pneumoniae*, *Staphylococcus aureus*, *Pseudomonas aeruginosa*, *Haemophilus influenzae*, *Escherichia coli*, *Acinetobacter baumannii*, and *Klebsiella oxytoca* with frequencies of 44 (28.0%), 39 (24.8%), 29 (18.5%), 22 (14.0%), 11 (7.0 %), 7 (4.5%), 3 (1.9%), and 2 (1.3%), respectively (Table 2). Additionally, all study participants were screened for TB, by Gene Xpert and *Mycobacterium tuberculosis* was detected in 39 (9.6%) of the participants. Among TB-positive participants, rifampicin-resistant was detected in 5 (12.8%) of the cases.

### 3.3. Antimicrobial Susceptibility Patterns of Bacterial Isolates

Among Gram-negative isolates, resistance to tetracycline, amoxicillin-clavulanate, co-trimoxazole, and chloramphenicol for *Klebsiella pneumoniae,* which was the most frequently isolated species, was 93.2%, 88.6%, 88.6%, and 79.5%, respectively. Whereas low resistance to ciprofloxacin (2.3%), cefuroxime (4.5%), piperacillin-tazobactam (4.5%), ceftazidime (6.8%), and amikacin (9.1%) was observed for *Klebsiella pneumoniae* isolates. *Pseudomonas aeruginosa* isolates showed more resistance to ceftazidime (63.6%) and gentamicin (54.5%), while *Haemophilus influenzae* isolates were more resistant to tetracycline (90.9%) and ampicillin (54.5%) (Table 3).

Among Gram-positive isolates, *Streptococcus pneumoniae*, the second most frequent isolate of all species, showed higher resistance to oxacillin (56.4%), penicillin (56.4%), and erythromycin (48.7%), while it showed lower resistance to clindamycin (10.3%), co-trimoxazole (12.5%), and ciprofloxacin (17.9%). *Staphylococcus aureus* resist more to tetracycline (86.2%) and co-trimoxazole (72.4%) (Table 4). Moreover, the prevalence of methicillin-resistant *Staphylococcus aureus* (MRSA) was found to be 34.5% (*n* = 10).

Multidrug resistance (MDR), resistance to 3 or more antimicrobials, was observed in 99 (63.1%) of the isolates, and high level of MDR was observed among *Klebsiella pneumoniae* (97.7%) and *Staphylococcus aureus* (89.7%), followed by *Pseudomonas aeruginosa* (45.5%) and *Haemophilus influenzae* (36.4%) (Table 4).

### 3.4. Factors Associated with Culture Positivity and Antimicrobial Resistance

Multivariable logistic regression indicated that aging (AOR = 2.43; 95% CI: 1.12–5.28, *p*=0.025), cigarette smoking (AOR = 4.67, 95% CI: 2.39–9.20, *p* value <0.001), and alcohol consumption (AOR = 5.58, 95% CI: 3.14–9.92, *p* value <0.001) were significantly associated with culture positivity (Table 5).

## 4. Discussion

The increased bacterial infection causing pneumonia and antimicrobial resistance become a serious public health concern. In our study, the overall prevalence of bacterial isolates was 38.7%, consistent with studies from Ethiopia reported by Temesgen et al. [1], Adhanom et al. [12], Regasa et al. [31, 32], and findings from Nigeria reported by Salami et al. [44]. However, the finding in this study was higher than the findings from Brazil, China, and the USA reported by Assunção et al. [5], Lin et al. [45], and Carugati et al. [46], respectively, while it was lower than the findings in other parts of Ethiopia and elsewhere [10, 21, 33, 34, 47–49]. The inconsistency might be explained as real prevalence variations or methodological differences.

Although pneumonia can be caused by a variety of bacterial species, some bacteria are frequent causes due to a variety of reasons. In the present study, the predominant bacterial isolates were *Klebsiella pneumoniae* (28.0%) and *Streptococcus pneumoniae* (24.8%). These two pathogens were reported as predominant in different studies [1, 10, 12, 50]. This predominance may be due to their capsular nature and the emergence of strains from both species that can acquire additional genetic traits [51, 52]. The prevalence of *Staphylococcus aureus* and methicillin-resistant *Staphylococcus aureus* (MRSA) in our study was 18.5% and 34.5%, respectively. This indicates that MRSA is becoming an important pneumonia-causing pathogen in the study area and supported by other studies [12, 53, 54].

In line with previously documented results, our finding revealed an increased prevalence of drug-resistant isolates that could possess a significant health risk. *Klebsiella pneumoniae* was resistant and sensitive to tetracycline and ciprofloxacin in 95.5% and 97.7%, respectively. It was comparable to the study conducted in Ethiopia by Temesgen et al. with 100% resistance and 96.7% sensitivity [1]. Moreover, the sensitivity of this pathogen to ciprofloxacin was comparable with a study conducted in China (91.7%) [47]. *Streptococcus pneumoniae*, the second most frequent species in our study, showed resistant to oxacillin in 56.4%, while only 10.3% of the isolates were resistant to clindamycin. This was comparable with studies conducted in Ethiopia [1, 34]. Moreover, *Staphylococcus aureus* was resistant to penicillin in 75.9%. Other studies reported comparable findings [1, 5, 12]. *Pseudomonas aeruginosa* was resistant to gentamicin in 59.1%, compared with other studies [1, 3, 5, 10, 34]. The possible explanation of drug resistance variations might be due to difference in distribution of resistance strains in different localities. The decreasing susceptibility might be due to increasing trend of using antibiotics.

The overall prevalence of multidrug-resistant (MDR) isolates in this study was 63.1% which was in agreement with findings reported from Ethiopia by Regasa et al. (56.7%) [34] and 54.8% [32]. In contrast, a study reported by Temesgen et al. identified higher prevalence (76.0%) [1], and lower results were reported from Mekelle, Ethiopia, by Adhanom et al. (17.9%) [12] and from China by Luan et al. (24.5%) [3] which could be due to different reasons [1, 12] among which poor drug quality, antibiotic prescribing differences such as misuse and/or incomplete treatment courses of antibiotics, overprescription due to a poor diagnostic set-up, and irrational drug use can be mentioned. In support of this, in this study, almost all cases (99%) were due to community-acquired pneumonia (CAP) and only 1% of them were due to hospital-acquired pneumonia (HAP).

Several studies reported aging as a risk factor for bacterial pneumonia. In the present study, the age group of >64 years was 2.4 times more likely to have bacterial pneumonia compared to the age group of 5–15 years [55]. Similar findings were reported from Spain [56, 57], Pakistan [49], Japan [58], and the USA [59]. The decline of the immune status in the older age may be the possible reason. On the other hand, other studies reported young age as a risk factor for the disease in Ethiopia [12] and Ghana [60]. This suggests that occurrence of high transmission due to crowded-living or presence of undernourishment weakens the immune system of the young population.

In this study, smoking increases the risk of bacterial pneumonia 4.7 times compared to those who were nonsmokers. Likewise, cigarette smoking, as a risk factor for pneumonia, was reported from Kenya [61] and Spain [55, 57]. This may be due to the fact that smoking decreases the number and, at the same time, the action of cilia facilitating the entry of microorganisms to the respiratory tract [61, 62].

Several studies also showed that alcohol consumption increases the risk of bacterial pneumonia. In our study, alcohol consumers (although this study did not identify the level of consumption) were 5.6 times more likely to have bacterial pneumonia compared to those who were nonconsumers. Similarly, other studies conducted in Ethiopia [1, 12], China [47], Spain [57], England [63], and Europe [62] identified alcohol consumption as a risk factor for acquiring bacterial pneumonia. This may be due to the fact that the sedative properties of alcohol minimize oropharyngeal tone that results in a high risk of aspiration of pathogens from the upper respiratory tract. Moreover, high levels of alcohol consumption can alter the alveolar macrophage function, hence withdrawing pulmonary defense against infection. Alcohol depresses cough, decreases endothelial adherence, lowers chemotaxis, and suppresses B cell and T cell spreading out which contributes to reduced clearance mechanism of lung cells [1, 12, 62, 63].

## 5. Conclusions

The overall prevalence of bacterial isolates, in this study, was 38.7%. This high prevalence needs expanding routine bacterial culture and antimicrobial susceptibility testing in the study area. The predominant isolates were *Klebsiella pneumoniae* (28.0%) and *Streptococcus pneumoniae* (24.8%). This indicates that there is an urgent need of strengthening vaccination practices for *Streptococcus pneumoniae.* The prevalence of multidrug-resistant (MDR) pathogens was 63.1%. The predominant isolate, *Klebsiella pneumonia,* was highly resistant to tetracycline in 95.5%, followed by penicillin and amoxicillin/clavulanic acid in 81.5% and 75.9%, respectively. These high figures, in general, recommend avoiding misuse, incomplete treatment courses, overprescription, and irrational use of antibiotics. Aging, cigarette smoking, and alcohol use were factors associated with culture positivity. Therefore, preventive measures to minimize the risk of the disease should include life-style factors such as smoking and alcohol use. Moreover, strengthening regular surveillance systems are essential for assessing predominant pathogens and antibiotic resistance patterns.

## Figures and Tables

**Table 1:** Sociodemographic characteristics and clinical data of pneumonia suspected patients, Dessie, 2020.

Characteristics	Frequency	Percentage (%)
Sex		
Male	221	54.4
Female	185	45.6

Age (in years)		
5–14	30	7.4
15–24	57	14.0
25–44	96	23.6
45–64	67	27.6
>64	156	38.4

Residence		
Urban	249	61.3
Rural	157	38.7

Educational level		
Unable to read and write	158	38.9
Read and write only	48	11.8
Primary education	56	13.8
Secondary education	67	16.5
College and above	77	19.0

Marital status		
Married	187	46.1
Single	107	26.4
Divorced	70	17.2
Widowed	42	10.3

Occupational status		
Employed	49	12.1
Unemployed	120	29.6
Farmer	161	39.7
Others	76	18.7

HIV		
Positive	43	10.6
Negative	383	89.4

TB		
Positive	39	9.6
Negative	385	90.4

Asthma		
Yes	23	6.0
No	383	94.0

Diabetes		
Yes	20	5.0
No	386	95.5

Hypertension		
Yes	13	3.0
No	393	93.0
**Total**	**406**	**100**

**Table 2:** The prevalence of bacterial isolates identified from pneumonia suspected patients, Dessie, 2020.

Bacterial isolates	Frequency	Percentage

Gram-positive (*n* = 68)		
*S. pneumoniae*	39	24.8
*S. aureus*	29	18.5
Gram-negative (*n* = 89)		
*K. pneumoniae*	44	28.0
*P. aeruginosa*	22	14.0
*H. influenzae*	11	7.0
*E. coli*	7	4.5
*A. baumannii*	3	1.9
*K. oxytoca*	2	1.3
Overall	**157**	**38.7**

**Table 3:** Antimicrobial resistance profile of bacterial isolates, Dessie, 2020.

GP isolates	Antimicrobials tested															
	E	OXA	TE	COT	DA	CN	P	CXT	C	CIP						
*S. pneumoniae* (*n* = 39)	19 (48.7)	22 (56.4)	16 (41.0)	5 (12.8)	4 (10.3)	NT	NT	NT	NT	NT						
*S. aureus* (*n* = 29)	14 (48.3)	NT	25 (86.2)	21 (72.4)	3 (10.3)	8 (27.6)	22 (75.9)	10 (34.5)	8 (27.6)	7 (24.)						
GN isolates	AMP	AMC	TZP	CRO	AK	CXM	CIP	COT	CN	IMP	GAZ	TE	c	PEP	MER	AZT
*K. pneumoniae* (*n* = 44)	44 (100)	39 (88.6)	2 (4.5)	6 (13.6)	4 (9.1)	2 (4.5)	1 (2.3)	39 (88.6)	7 (15.9)	6 (13.6)	3 (6.8)	42 (95.5)	35 (79.5)	NT	NT	NT
*P. aeruginosa* (*n* = 22)	NT	NT	2(9.1)	NT	2 (9.1)	NT	1 (4.5)	NT	12 (54.5)	1 (4.5)	14 (63.6)	NT	NT	2 (9.1)	NT	NT
*H. influenzae* (*n* = 11)	6 (54.5)	0 (0)	NT	4 (36.4)	NT	NT	4 (36.4)	NT	NT	NT	NT	10 (90.9)	NT	NT	0 (0)	0 (0)
*E. coli* (*n* = 7)	5 (71.4)	3 (42.9)	1 (14.3)	1 (14.3)	1 (14.3)	1 (14.3)	0 (0)	5 (71.4)	1 (14.3)	1 (14.3)	1 (14.3)	5 (71.4)	5 (71.4)	NT	NT	NT
*A. baumannii* (*n* = 3)	NT	NT	2 (66.7)	NT	1 (33.3)	NT	2 (66.7)	2 (66.7)	2 (66.7)	NT	2 (66.7)	2 (66.7)	NT	2 (66.7)	1 (33.3)	NT
*K. oxytoca* (*n* = 2)	2 (100)	1 (50)	0 (0)	0 (0)	0 (0)	1 (50)	0 (0)	1 (50)	0 (0)	0 (0)	0 (0)	2 (50)	2 (100)	NT	NT	NT

Note: GP, Gram-positive; GN, Gram-negative; E, erythromycin; OXA, oxacillin; DA, clindamycin; P, penicillin; CXT, cefoxitin; AMP, ampicillin; AMC, amoxicillin/clavulanate; TZP, piperacillin/tazobactam; CRO, ceftriaxone; AK, amikacin; CXM, cefuroxime; CIP, ciprofloxacin; COT, co-trimoxazole; CN, gentamicin; IMP, imipenem; CAZ, ceftazidime; TE, tetracycline; C, chloramphenicol; PEP, cefepime; MER, meropenem; AZT, aztreonam; NT, not tested (based on CLSI-2019 Guideline).

**Table 4:** Multidrug resistance (MDR) profile of the isolated organisms, Dessie, 2020.

Bacterial isolates (*n*)	Degree of resistance									
	R0 (%)	R1 (%)	R2 (%)	R3 (%)	R4 (%)	R5 (%)	R6 (%)	R7 (%)	R8 (%)	Total MDR (≥R3)

*K. pneumoniae* (*n* = 44)	0 (0)	0 (0)	1 (2.3)	5 (11.4)	5 (11.4)	16 (36.4)	11 (25.0)	4 (9.1)	2 (4.5)	43 (97.7)
*S. pneumoniae* (*n* = 39)	01 (2.6)	15 (38.5)	19 (48.7)	2 (5.1)	2 (5.1)	0(0)	0(0)	0(0)	0(0)	4 (10.3)
*S. aureus* (*n* = 29)	0 (0)	0 (0)	3 (10.3)	4 (13.8)	12 (41.4)	5 (17.2)	5 (17.2)	0(0)	0(0)	26 (89.7)
*P. aeruginosa* (*n* = 22)	0 (0)	5 (22.7)	7 (31.8)	6 (27.3)	2 (9.1)	2 (9.1)	0(0)	0(0)	0(0)	10 (45.5)
*H. influenzae* (*n* = 11)	1 (9.1)	5 (45.5)	1 (9.1)	0(0)	4 (36.4)	0(0)	0(0)	0(0)	0(0)	4 (36.4)
*E. coli (n* *=* *7*)	0 (0)	0(0)	0(0)	2 (28.6)	2 (28.6)	2 (28.6)	0(0)	0(0)	1 (14.3)	7 (100)
*A. baumannii* (*n* = 3)	0 (0)	0(0)	0(0)	0(0)	0(0)	1 (33.3)	2 (66.7)	0(0)	0(0)	3 (100)
*K. oxytoca* (*n* = 2)	0 (0)	0(0)	0(0)	0(0)	1(50)	1(50)	0(0)	0(0)	0(0)	2 (100)

Note: R0, susceptible to all antibiotics; R1–R8, resistance to 1, 2, 3, 4, 5, 6, 7, and 8 antibiotics; ≥R3, resistance to 3 or more antibiotics; MDR, multidrug resistance.

**Table 5:** Multivariable logistic regression analysis of culture positivity identified from pneumonia suspected patients, Dessie, 2020.

Variables	Culture positivity		COR (95% CI)	*p* value	AOR (95% CI)	*p* value
	Positive, *N* (%)	Negative, *N* (%)				
Sex						
Female	59 (32)	126 (68)	1		1	
Male	98 (44)	123 (56)	1.70 (1.13–2.56)	0.011	0.87 (0.44–1.72)	0.678

Age (years)						
5–15	3 (10)	27 (90)	1		1	
15–24	13 (23)	44 (77)	2.70 (0.68–10.19)	0.154	0.12 (0.02–0.69)	0.017
25–44	20 (21)	76 (79)	2.40 (0.65–8.61)	0.190	0.27 (0.10–0.73)	0.010
44–64	43 (64)	24 (36)	16.13 (4.43–58.76)	<0.001	0.35 (0.17–0.75)	0.006
>64	78 (50)	78 (50)	9.00 (2.62–30.90)	<0.001	2.43 (1.12–5.28)	0.025

Residence						
Urban	100 (40)	149 (60)	1		1	
Rural	57 (36)	100 (64)	0.83 (0.55–1.25)	0.365	1.04 (0.42–2.59)	0.941

Smoking						
No	67 (24)	208 (76)	1		1	
Yes	90 (69)	41 (31)	6.62 (4.19–10.47)	<0.001	4.67 (2.39–9.20)	<0.001

Alcohol drinking						
No	104 (66)	54 (34)	1		1	
Yes	53 (21)	195 (79)	6.54 (4.20–10.20)	<0.001	5.58 (3.14–9.92)	<0.001

## Data Availability

The datasets used to support the findings of this study are available from the corresponding author upon request.

## Abbreviations

APHI: Amhara Public Health Institute
ARDS: Acute respiratory distress syndrome
ART: Antiretroviral treatment
AST: Antimicrobial susceptibility test
ATCC: American Type Culture Collection
BAP: Blood agar plate
BMI: Body mass index
CAP: Community-acquired pneumonia
CHO: Chocolate agar
CLSI: Clinical and Laboratory Standards Institute
COPD: Chronic obstructed pulmonary disease
ESBL: Extended spectrum beta-lactamase
HCAP: Hospital-acquired pneumonia
MAC: MacConkey agar
MDR: Multidrug resistance
MHA: Muller–Hinton agar
MIC: Minimum inhibitory concentration
MRSA: Methicillin-resistant *Staphylococcus aureus*
MSA: Mannitol salt agar
MUAC: Mid-upper arm circumferences
NAD: Nicotinamide adenine dinucleotide
PCR: Polymerase chain reaction
PCV: Pneumococcal conjugate vaccine.
